# Occurrence and suppression effect of Otoacoustic Emissions in normal hearing adults with tinnitus and hyperacusis

**DOI:** 10.1590/S1808-86942012000100014

**Published:** 2015-10-20

**Authors:** Daila Urnau, Tania Maria Tochetto

**Affiliations:** a Master's degree in human communication disorders (UFSM) (speech therapist); b Doctoral degree in the science of human communication disorders (speech therapist, associate professor of the Speech Therapy Department, UFSM, Santa Maria, RS)

**Keywords:** acoustic stimulation, efferent pathways, hyperacusis, tinnitus

## Abstract

The association between tinnitus and hyperacusis is common according to the literature.

**Aim:**

To verify the occurrence and the suppression effect of transient otoacoustic emissions (TEOAE), the existence of association between tinnitus degrees and hyperacusis degrees, and between the suppressive effect of TEOAE and laterality, tinnitus and hyperacusis degrees in normal hearing adults with complaints of tinnitus and hyperacusis.

**Materials and Methods:**

25 normal hearing subjects with complaints of hyperacusis and tinnitus were studied in this cross-sectional study. The Tinnitus Handicap Inventory (THI) was used for the classification of tinnitus degrees, and the Loudness Discomfort Level (LDL) for the hyperacusis classification.

**Results:**

The occurrence of TEOAE ranged from 33 to 88%. We observed the presence of TEOAE suppression effect on 63.7% in the right ear and 81.7% in the left ear. There was no significant correlation between the degrees of tinnitus and hyperacusis in both ears. No statistically significant associations between the TEOAE suppression effect and laterality, tinnitus degrees and hyperacusis degrees were found.

**Conclusion:**

The occurrence of TEOAE was lower than that found in normal hearing adults. A higher percentage of the presence of TEOAE suppression effect has been found in both ears. No association between the variables was observed.

## INTRODUCTION

Tinnitus (acuphenes) may be characterized by conscious auditory perception of a sound that originates in one or both ears, the head, or without a specific site, in the absence of an external sound stimulus^1^, ^2^.

Questionnaires are useful for evaluating these patients because there are no objective methods for detecting tinnitus and establishing its severity^3^. The Tinnitus Handicap Inventory (THI) is one of the most commonly accepted methods for assessing tinnitus^4^.

Hyperacusis is decreased tolerance to sound^5^; it appears to be a pre-tinnitus state an early indicator of susceptibility to tinnitus^6^.

The basic evaluation of hyperacusis comprises a detailed clinical history, pure tone audiometry, immittance testing, and investigation of the loudness discomfort level^7^.

The association between tinnitus and hyperacusis ranges from 63% to 90% in the literature^8^, ^9^, ^10^, ^11^. These phenomena appear to have a common physiologic and pathologic basis, as both are related with the efferent auditory system^12^.

Efferent fibers from several points of the central nervous system gather in the superior olivary complex. Ipsilateral and contralateral efferent fibers emerge from this complex to the human cochlea; this is the olivocochlear bundle, which consists of a lateral and a medial system. The lateral bundle consists of non-myelinated fibers that project ipsilaterally from the lateral portion of the superior olivary complex to the inner hair cells. The medial bundle consists of myelinated fibers that project ipsilateral and contralaterally from the medial portion of the superior olivary complex to the outer hair cells (OHC)^13^, ^14^. Thus, the mechanical movement of the OHC is controlled by the medial olivocochlear system, which Rasmussen described in 1946^15^.

Otoacoustic emissions (OAE) are sound produced in the cochlea, and are detected in the outer ear canal; these emissions are a recording of the mobility and mechanical capacity of OHC^16^.

Contralateral noise has an inhibitory effect on OHC, which reduces the amplitude of OAE^17^, ^18^. Several studies of normal-hearing subjects^19^, ^20^ have demonstrated this phenomenon, know as OAE suppression; it is evidence that the medial olivocochlear system, which innervates the OHC, is intact.

Based on the assumption that the medial olivocochlear system modulates OHC movement by the medial olivocochlear tract, any malfunction of this system could generate hyperacusis and tinnitus, which demonstrates their apparent connection.

The purpose of this study was to verify the occurrence and transient otoacoustic emissions (TOAEs) suppression, the existence of associations between degrees of tinnitus and hyperacusis, and between TOAE suppression and handedness, and between degrees of tinnitus, and hyperacusis in normal-hearing subjects that complained of tinnitus and hyperacusis.

## MATERIAL AND METHODS

A cross-sectional, descriptive, non-experimental, quantitative study was made of data gathered from normal-hearing subjects that complained of hyperacusis and tinnitus.

Data were gathered from May to July 2010. Subjects that agreed to participate and who signed a free informed consent form after receiving information about the purpose and methods of this study were enrolled (Resolution 196/1996).

This study is part of the project “Effect of suppressing otoacoustic emissions” that had been approved by the institutional review board (no. 23081.010072/2008-73).

Auditory complaints – hyperacusis and tinnitus – were investigated in the clinical history.

Only normal-hearing subjects were enrolled in this study: the threshold in pure tone audiometry had to be not more than 25 dB at all frequencies^21^; the tympanogram had to be type A^22^, and acoustic reflexes had to be present.

Pure tone audiometry was done at 250, 500, 1000, 2000, 3000, 4000, 6000, and 8000 Hz (air conduction), and at 500, 1000, 2000, 3000, and 4000 Hz (bone conduction). A two-channel digital audiometer (Fornix^®^, model FA-12 type I) and over-the-ear phones (Telephonics^®^, TDH-39P).

A middle ear analyzer (Interacoustics^®^, AZ7), earphones (TDH-39), and pad (MX-41) were used for tympanometry and to measure the acoustic reflex; the probe tone was 220 Hz at 70 dBHL for tympanometry, calibrated according to the ISO 389-1991 norm.

There were 25 subjects, 16 male and 9 female, aged from 21 to 70 years.

The Tinnitus Handicap Inventory, adapted into Brazilian Portuguese, was applied to classify the degree of tinnitus. This questionnaire consists of 25 questions that evaluate the emotional, functional, and catastrophic aspects of tinnitus^23^. Each question has three answers: yes (4 points), sometimes (2 points), and no (no point). The scores are added up and used to classify tinnitus in degrees, as follows: slight (0 to 16), mild (18 to 36), moderate (38 to 56), severe (58 to 76), and catastrophic (78 to 100)^24^.

The authors translated and applied a questionnaire on manual handedness, “The assessment and analysis of handedness: The Edinburgh inventory”^25^. It contains questions on manual preference for several activities of daily life. The score is used to classify subjects as right-handed, left-handed, or ambidextrous. The latter were not enrolled because they do not have a defined handedness^26^.

The Loudness Discomfort Level (LDL) was measured from 250 to 8000 Hz by the same pure tone audiometry device. Starting with pure tone thresholds, the intensity of a pure pulsed tone was gradually increased in 10 dB steps until reaching 60 dB and thereafter in 5 dB steps until the subject reported discomfort with this sound, but before it was perceived as painful. Each stimulus lasted about two seconds, with an approximately one second interval.

Hyperacusis was classified as negative, mild, moderate, and severe according to the discomfort level. Negative hyperacusis was negative when the LDL was 95 dB or higher at all frequencies; it was mild when the LDL was 80 to 90 dB at two or more frequencies; it was moderate when the LDL was 65 to 75 dB at two or more frequencies; and it was severe when the LDL was 60 dB or less at two or more frequencies^27^.

TOAEs were recorded in an acoustic booth with a Smart EP USB Jr. device (Intelligent Hearing Systems). TOAEs were analyzed at 1000 to 4000 Hz; 80 dBSPL clicks lasting about 19 seconds were used. The signal-to-noise ratio was at least 6 dB. OAEs were measured to assess the presence of OAE suppression with and without noise in the contralateral ear.

White noise generated by the audiometer above was used as the suppressing acoustic stimulus, at 60 dBHL through TDH-39 P earphones. The phone was adapted over the contralateral ear at the beginning of the test to avoid handling the OAE probes.

OAE suppression was calculated by subtracting the OAE response amplitude without an acoustic stimulus from the OAE response with an acoustic stimulus. Zero or negative values indicated non-suppression of OAEs; positive values indicated suppression. A more positive suppression effect correlates with a higher activity of the medial olivocochlear system^28^, ^29^, ^30^.

TOAE suppression was considered as present when it manifested in most of the frequencies at which TOAEs were found.

The results of severe and catastrophic tinnitus were grouped with the moderate tinnitus results, and the slight and mild tinnitus results were also grouped together, in both cases for the statistical analysis. Negative and mild hyperacusis were groups, as were the moderate and severe hyperacusis levels for the same purpose.

The results were tabulated and analyzed statistically by applying Fisher's exact test and Spearman's correlation coefficient. The statistical significance level was 5% (*p*<0.05).

## RESULTS

### TOAEs – occurrence and suppression

**Figure 1:**
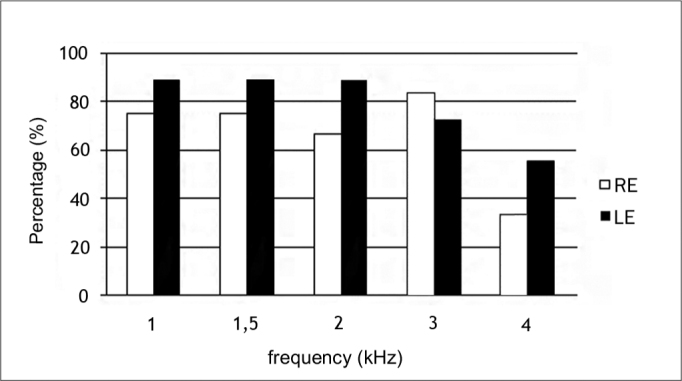
Occurrence of TOAE per frequency range in normal-hearing subjects with complaints of hyperacusis and tinnitus per frequency range. RE – right ear. LE – left ear

Figure 1 shows the occurrence of TOAEs per frequency. TOAEs occurred more often in the left ear at all frequencies except at 3 KHz.

Table 1 shows the results of TOAE suppression per frequency in the right and left ears of normal-hearing subjects with hyperacusis and tinnitus.

**Table 1:** Occurrence of TOAE suppression per frequency range in right and left ears of normal-hearing subjects with tinnitus and hyperacusis.

Frequency	Right ear	Left ear
1000 Hz		

Suppression	11(61.1%)	13(81.2%)
No suppression	7 (38.9%)	3 (18.8%)

1500 Hz		

Suppression	13 (72.2%)	14 (87.5%)
No suppression	5 (27.8%)	2 (12.5%)

2000 Hz		

Suppression	8 (50%)	13 (81.2%)
No suppression	8 (50%)	3 (18.8%)

3000 Hz		

Suppression	14 (70%)	9 (69.2%)
No suppression	6 (30%)	4 (30.8%)

4000 Hz		

Suppression	5 (62.5%)	9(90%)
No suppression	3 (37.5%)	1 (10%)

The suppression effect was present in 63.7% of cases in the right ear, and in 81.7% in the left ear at all frequencies for each ear.

Although there was more suppression in the left ear compared to the right ear, there was no statistically significant association between TOAE suppression and ear, at all frequencies (*p*>0.05).

**Figure 2:**
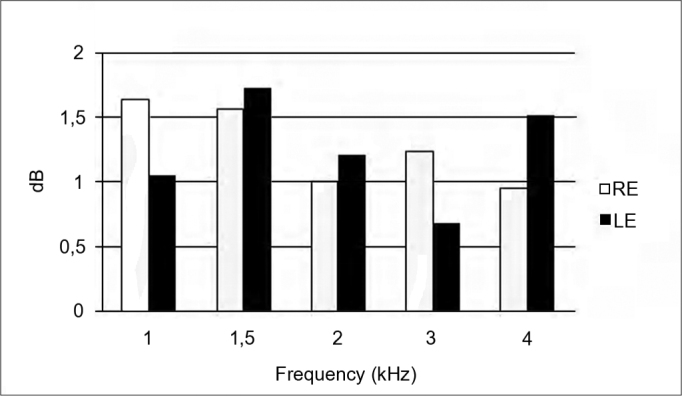
Mean TOAE suppression amplitude per frequency range in the right and left ears. RE – right ear. LE – left ear.

Figure 2 shows the mean suppression amplitudes at 1, 1.5, 2, 3, and 4 KHz in right and left ears. The mean suppression in the right ear was 1.28 dB; in the left ear it was 1.25 dB.

The highest mean amplitude suppression in the left ear occurred at 1.5, 2, and 4 KHz. The amplitude was higher in the right ear at 1 KHz and 3 KHz compared to the left ear (Figure 2).

### Degrees of tinnitus in the THI and hyperacusis grades in the LDL

Spearman's correlation coefficient revealed no significant correlation between the degrees of tinnitus (THI) and of hyperacusis (LDL) in the right ear (r=0.24; *p*=0.27) and left ear (r=-0.04 *p*=0.86).

### Handedness and TOAE suppression

Right-handed subjects comprised 84% of the sample; 12% were left-handed, and 4% were ambidextrous. There was no statistically significant association between handedness and the occurrence of TOAEs suppression in right and left ears (Tables 2 and 3).

**Table 2:** Handedness and occurrence of TOAE suppression in the right ear.

Handedness	TOAE suppression	
			Present	Absent
Right-handed	16 (84.2%)	3 (15.8%)
Left-handed	1 (33.3%)	2 (66.7%)

Fisher's exact test (*p*= 0.12).

**Table 3:** Handedness and occurrence of TOAE suppression in the left ear.

Handedness	TOAE suppression	
	Present	Absent
Right-handed	16 (94.1%)	1 (5.9%)
Left-handed	1 (100%)	0 (0%)

Fisher's exact test (*p*= 1.0).

### THI and TOAE suppression

The degrees of tinnitus in the THI were: mild (44%), moderate (24%), slight (20%), severe (8%), and catastrophic (4%). The mean sum of THI results was 322 (standard deviation – 209).

There was no statistically significant association between the degrees of tinnitus as measured by the THI and the occurrence of TOAE suppression in right and left ears (Tables 4 and 5).

**Table 4:** Degrees of tinnitus and occurrence of TOAE suppression in the right ear.

Degrees of Tinnitus (THI)	TOAE suppression	
	Present	Absent
Slight and Mild	11 (73.3%)	4 (26.7%)
Moderate, Severe, and Catastrophic	6 (75%)	2 (25%)

Fisher's exact test (*p*=1.0).

**Table 5:** Degrees of tinnitus and occurrence of TOAE suppression in the left ear.

Degrees of Tinnitus (THI)	TOAE suppression	
	Present	Absent
Slight and Mild	10 (100%)	0 (0%)
Moderate, Severe, and Catastrophic	7 (87.5%)	1 (12.5%)

Fisher's exact test (*p*=0.44).

### Hyperacusis grades (LDL) and TOAE suppression

**Figure 3:**
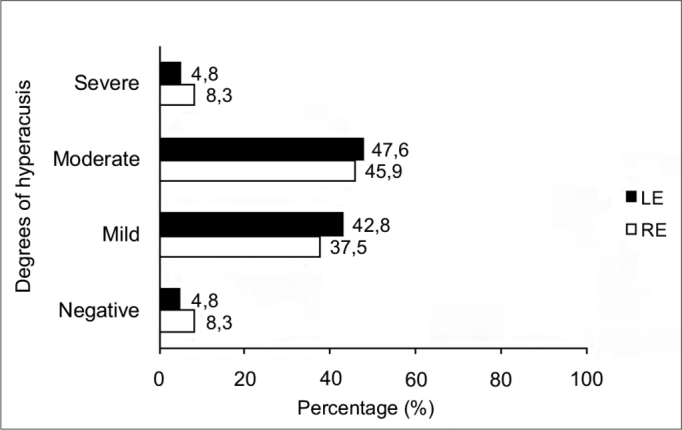
Degrees of hyperacusis based on the LDL in right and left ears of normal-hearing subjects with complaints of hyperacusis and tinnitus. RE – right ear. LE – left ear.

Figure 3 shows the degrees of hyperacusis based on the LDL, in the right and left ears.

There was no statistically significant association between degrees of hyperacusis as measured by the LDL and the occurrence of TOAE suppression in right and left ears (Tables 6 and 7).

**Table 6:** Degrees of hyperacusis based on the LDL and occurrence of TOAE suppression in the right ear.

Degrees of Hyperacusis (LDL)	TOAE suppression	
	Present	Absent
Negative and Mild	8 (72.7%)	3 (27.3%)
Moderate and Severe	11 (91.7%)	1 (8.3%)

Fisher's exact test (*p*=0.32).

**Table 7:** Degrees of hyperacusis based on the LDL and occurence of TOAE suppression in the left ear.

Degrees of hyperacusis (LDL)	TOAE suppression	
	Present	Absent
Negative and Mild	8 (100%)	0 (0%)
Moderate and Severe	9 (90%)	1 (10%)

Fisher's exact test (*p*=1.0).

## DISCUSSION

The occurrence rates of TOAEs ranged form 33% to 88% in this study of normal-hearing subjects with tinnitus and hyperacusis (Figure 1). TOAEs are found in 98% of normal-hearing individuals^31^. Other studies^28^, ^30^ have also found a lower rate of TOAE occurrence in patients with tinnitus compared to tinnitus-free individuals. Anatomical abnormalities of the outer ear canal or the middle ear (in this study all subjects had type A tympanometry curves and the acoustic reflexes were present), issues with equipment, and noise are some of the factors that may explain absence of TOAEs^32^.

There was no statistically significant association between the occurrence of TOAE suppression and the ear, at all frequencies. There were higher percentage rates of suppression in the left ear at all frequencies except for 3 KHz. Suppression was significantly higher in the right ear of in normal-hearing right-handed individuals at 1, 2, 3 e 4 KHz^33^.

The mean TOAE suppression values in the right and left ears were 1.28 and 1.25 dB respectively. Aita^8^ found similar mean TOAE suppression values (1.29 dB in the right ear, and 1.26 dB in the left ear) in subjects with hyperacusis. Mor & Azevedo^30^ found higher mean TOAE suppression rates in the right ear (2.6 dB) and lower suppression rates in the left ear (0.7 dB) in individuals with tinnitus and no hearing loss, compared to our mean findings.

The common finding in the three studies was a higher mean suppression in the right ear compared to the left ear. As most of the subjects in this study were right-handed, the likely explanation is handedness of the medial olivocochlear system. Studies have shown that the medial olivocochlear tract may be involved in maintaining a peripheral asymmetric pattern by which the cortex may modulate cochlear function^33^.

However, if we consider that the mean TOAE suppression was slightly higher in the right ear and that higher percentages of TOAE suppression occurred in the left ear (Table 1), the hypothesis of hemispheric predominance probably influenced by the medial olivocochlear system^33^, ^34^ was not confirmed. A high rate of TOAEs in the left ear (Figure 1) compared to the right may have influenced the high suppression rates in this ear.

The medial olivocochlear system has an inhibitory modulation effect on the rapid contractions of the OHC by causing slow contraction in these cells, thereby attenuating the cochlear amplification process^35^. This system may be activated by electric, chemical, or noise stimulation, thereby inhibiting OHC contraction and decreasing OAEs amplitude^36^. Thus, absence of OAE suppression, as evidenced by increased OAE amplitude, suggests abnormalities in the medial olivocochlear system.

TOAE suppression was 63.7% in the right ear and 81.7% in the left ear; thus, there was a higher percentage of reduction than increase in TOAE amplitude with contralateral noise. The literature, however, shows that TOAEs should present significantly higher amplitude values in the presence of contralateral noise in subjects with tinnitus and/or hyperacusis compared to individuals with no auditory complaints^14^, ^19^. Studies have demonstrated higher amplitudes after contralateral stimulation in subjects with hyperacusis^37^ and tinnitus^30^, ^38^, ^39^ compared to individuals with no auditory complaints.

Paglialonga et al.^40^ have suggested that TOAE suppression and amplitude evaluations may not be so sensitive to detect likely subclinical abnormalities in OHC function, and that distortion product otoacoustic emissions (DPOAEs) may be more sensitive. DPOAEs yield information per frequency, and may therefore be more sensitive than TOAEs in detecting OHC dysfunction in specific areas of the cochlea. On the other hand, TOAE suppression and amplitude yield broad band information – global measures of OHC integrity and function throughout the cochlea and the medial olivocochlear system.

Our findings showed a predominance of presence of TOAE suppression (63.7% and 81.7%). Similarly, Aita^8^ found lower amplitudes in the presence of contralateral noise in subjects with hyperacusis compared to the group without auditory complaints.

Most studies^30^, ^35^ of subjects with hyperacusis or tinnitus have reported a relationship between the presence of these complaints and reduced effectiveness of the medial olivocochlear system; we did not find this relationship in our study.

Conversely, different degrees of function between OHC and inner hair cells (IHC) may generate abnormal stimulation of cells in the dorsal cochlear nucleus, thereby causing tinnitus associated with neuron activity. Such a dysfunction may occur in the presence of partially injured OHC and normally functioning IHC, without altering the audiometric thresholds^12^.

There was not statistically significant association between degrees of tinnitus (THI) and hyperacusis (LDL) in both ears. These two methods tend to be used for one of these complaints only. There are published studies correlating the THI and other tinnitus assessments, such as the visual-analog scale (VAS)^4^ and Beck's Depression Inventory^31^.

Analysis of handedness revealed no statistically significant associations with the occurrence of TOAE suppression in each ear (Tables 2 e 3).

The predominance of one cerebral hemisphere over the other is well established^33^; it is thought that the medial olivocochlear system follows suit, and that there are higher suppression values in the right ear of right-handed individuals^34^. Fávero et al.^33^ have reported that the medial olivocochlear system operates causing a functional predominance of the right ear over the left ear in right-handed individuals; however, it does not appear to do so regularly throughout the cochlea.

The THI (Tinnitus Handicap Inventory) was created by Newman et al.^41^; it consists of 25 questions that aim to characterize and quantify tinnitus. It has been validated and summarized, it is reliable, and is easy to apply and interpret in the clinical setting. The THI deals with several effects of tinnitus on the quality of life of patients: functional reactions to tinnitus (concentration difficulty, and antisocial tendencies), emotional reactions to tinnitus (such as anger, frustration, depression), and catastrophic reactions to tinnitus (despair, sensation of severe disease, powerlessness)^41^.

In this study, the mean value of THI results was 32.2; the mild degree of tinnitus was the most frequent (score from 18 to 36). Other studies of normal-hearing individuals^40^, ^42^ have found a similar prevalence when using the THI. Figueiredo et al.^31^ and Pinto et al.^43^, however, have reported a higher rate of the moderate degree (the mean THI results were respectively 45.5 and 39) when using the same questionnaire. These studies were done of subjects that manifested hearing loss and tinnitus.

Sanchez et al.^44^ compared the clinical features of tinnitus and its effect on activities of daily life in subjects with and without hearing loss. The clinical features of tinnitus were similar in both groups, but interferences on concentration and emotional balance were significantly lower in the normal-hearing group, which may explain the presence of lower degrees in our study of normal-hearing subjects.

Normal-hearing individuals without otologic complaints had LDL values ranging from 86 to 98 dB HL at 0.5 to 8 KHz^45^.

The most frequent degree of hyperacusis, as measured by the LDL, was the moderate degree (discomfort level of 65 to 75 dB at two or more frequencies) in both ears (Figure 1). Ribeiro et al.^46^ found a higher percentage rate of the mild degree (61%); the mean LDL value was 82.5 dB in individuals with hyperacusis. Our data suggest that the presence of tinnitus with hyperacusis may reduce the tolerance of external sounds.

There was no statistically significant association between the degrees of tinnitus and hyperacusis and the occurrence of TOAE suppression (Tables 4, 5, 6, and 7). There are no reports in the literature on associations between the degrees of tinnitus as measured by the THI or the degrees of hyperacusis as measured by the LDL and the occurrence of EOA suppression.

## CONCLUSION

The occurrence of TOAEs in normal-hearing subjects with tinnitus and hyperacusis was lower compared to that in normal-hearing individuals without these symptoms.

There was a higher percentage rate of TOAE suppression in both ears, predominating in the left ear compared to the right ear of normal-hearing adults with tinnitus and hyperacusis.

There was no correlation between the degrees of tinnitus and hyperacusis. There were no associations among TOAE suppression, handedness, degrees of tinnitus and hyperacusis in normal-hearing subjects with tinnitus and hyperacusis.
